# Osseointegrated total hip replacement connected to a lower limb prosthesis: a proof-of-concept study with three cases

**DOI:** 10.1186/s13018-016-0348-3

**Published:** 2016-01-19

**Authors:** Aditya Khemka, Chalak I. FarajAllah, Sarah J. Lord, Belinda Bosley, Munjed Al Muderis

**Affiliations:** School of Medicine, University of Notre Dame Australia, Sydney, Australia; Norwest Private Hospital, Sydney, Australia; Rural Clinical School, University of Notre Dame Australia, Sydney, Australia; The Australian School of Advanced Medicine, Macquarie University, Sydney, Australia; National Health and Medical Research Council (NHMRC) Clinical Trials Centre, The University of Sydney, Sydney, Australia

**Keywords:** Osseointegration, Joint replacement, Transfemoral, Amputee

## Abstract

**Background:**

Osseointegrated implants are a suitable alternative for prosthetic attachment in individuals with a transfemoral amputation, who are unable to wear a socket. However, the small bone-implant contact area, reduced muscular leverage, and osteoporosis contraindicate osseointegrated implant use in transfemoral amputees with osteoporosis and a short residuum. We report on the feasibility of combining total hip replacement (THR) with an osseointegrated implant for prosthetic attachment.

**Methods:**

We retrospectively reviewed the cases of three transfemoral amputees who underwent osseointegration with THR between 2013 and 2014. In a two-stage procedure, a custom-made femoral prosthesis was connected to a THR with a modular revision stem and a stoma was created. Clinical outcomes were assessed at baseline and 1.5–2.5-year follow-up using standard measures of health-related quality of life, ambulation, and activity levels including the Short Form-36 (SF-36), Questionnaire for Transfemoral Amputees (Q-TFA), Timed Up and Go test, and 6-min walk test.

**Results:**

Patient age ranged from 35 to 65 years. There were no major adverse events, but there was one case of superficial infection. All patients showed improved Q-TFA and SF-36 scores. Two patients who were wheelchair-bound at baseline became community ambulators, and the third patient exhibited improved ambulation.

**Conclusions:**

This study demonstrated the feasibility of combining a THR with an osseointegrated implant in transfemoral amputees.

## Background

Traditionally, transfemoral amputees have been rehabilitated using a socket prosthesis. However, over the past two decades, innovative surgical techniques that connect the lower limb prosthesis directly to the bone via osseointegration have emerged. These techniques were developed to overcome reductions in amputee quality of life, which occur secondary to socket-residuum interface problems including significant discomfort, poor fit, lack of rotational control, reduced proprioception, and associated energy loss due to prosthetic pistoning [1–3]. The structural and functional connection between the macroporous surface of metal implants and living bone [4] is achieved by either bone on-growth (screw fixation implants) [5] or bone penetration and in-growth (press-fit implants) [6].

Previous studies have reported the benefits of osseointegrated implants in transfemoral amputees. These included improvements in quality of life [5], prosthetic use [7], body image [8], hip motion range [9], sitting comfort [10], donning and doffing [11], osseoperception [12], and walking ability [13, 14]. Fewer studies have reported on the safety of osseointegrated implants in transfemoral amputees. Two studies in amputees with screw-type implants reported that superficial infections occurred in half of the study population but that there was a low risk of deep infection leading to implant removal (<3 %) [5, 15]. A prospective multicentre study in amputees with press-fit implants reported that there were no implant removals secondary to infection and that the rate of superficial infections was lower (<30 %), when compared with amputees with screw-type implants [16].

Transfemoral amputees with a short femoral residuum represent a challenge for rehabilitation, using not only sockets but also osseointegrated implants. In a recent study on prosthetic rehabilitation for amputee veterans in the USA, the degree of amputation was significantly associated with the prosthetic prescription, with >80 % of the transfemoral amputees failing to receive a prosthesis in the first year after amputation [17]. The prosthetic function in such patients is severely compromised because of limited muscle attachment and a small surface area for force distribution [18]. Finding an appropriate socket alignment and fit can be difficult and could lead to a substantial proportion of patients becoming wheelchair users [19].

The use of osseointegrated implants for transfemoral amputees with a short residuum presents several challenges. Biomechanical studies have shown that a small bone-implant contact area decreases the likelihood of a structural union, thereby resulting in an increased risk of aseptic loosening [20, 21]. Furthermore, interface load depends on the degree of amputation; in those with a shorter residuum, there is an abnormal increase in force moments during normal gait and falling [22]. A recent comparative study indicated that transfemoral amputees had significantly reduced bone mineral density (BMD) at the hip and femoral residuum, compared with the BMD in the intact contralateral limb [23]. Such osteoporosis increases the risks of aseptic loosening and fragility fractures of the hip. Likewise, in older amputees with concomitant ipsilateral hip arthritis, it has been hypothesized that the biomechanical forces could aggravate arthritic symptoms [24].

Therefore, alternative implant concepts are required to overcome these biomechanical challenges. One innovative idea is to convert the proximal joint to weight sharing rather than weight bearing by involving it in implant fixation by way of a joint replacement. We recently utilized this concept by combining total knee replacement with an osseointegrated implant in transtibial amputees who were not eligible for standard osseointegrated implants because of a short residuum or the presence of knee arthritis [25]. Based on that successful experience, we proposed to develop this concept further and adapt it for transfemoral amputees with a short femoral residuum.

The aim of this paper was to describe the surgical technique and midterm results of combining a total hip replacement (THR) with an osseointegrated implant for prosthetic rehabilitation in transfemoral amputees.

## Methods

### Patients

**Fig. 1:**
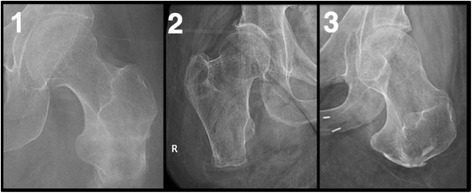
Radiographs of the residual bones for Case 1, 2 and 3

We retrospectively reviewed the cases of three individuals with transfemoral amputations who underwent an osseointegrated implant combined with a THR at our centre. Eligible patients included those with short transfemoral amputations (<10 cm) who presented with socket-related problems and who had arthritis with or without severe osteoporosis (Fig. 1). All patients were treated by the investigators at the specialized orthopaedic osseointegration clinic. All subjects provided written informed consent.

### Surgical technique

The two-stage surgical procedure was performed with 5–8 weeks in between the procedures. An osseointegrated femoral prosthesis was connected to the custom-made THR prosthesis to transfer the load directly to the pelvis through the acetabulum. This allowed the femoral residuum and hip joint to act as weight-sharing rather than weight-bearing structures.

### Implant design

**Fig. 2:**
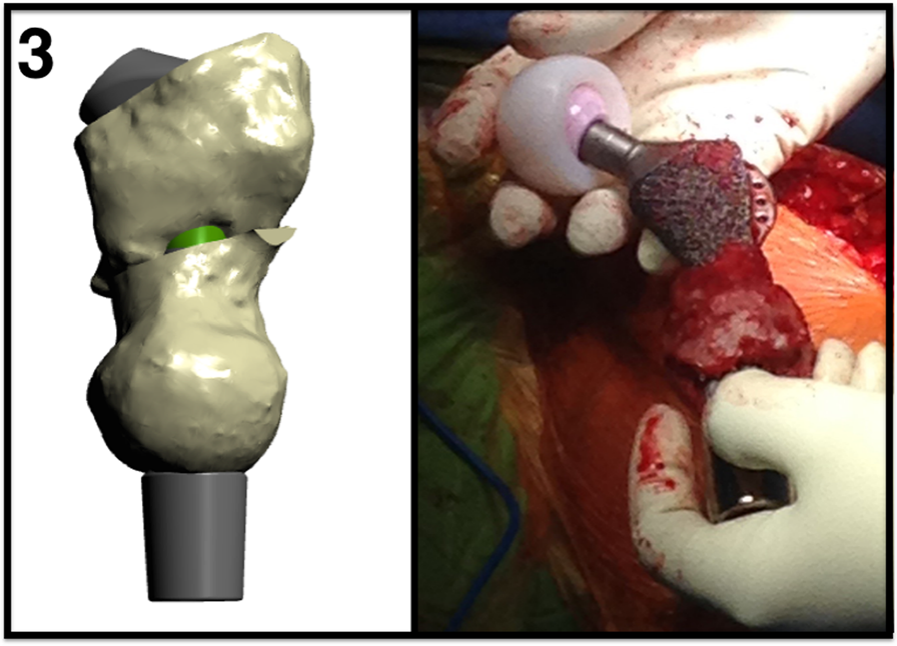
Representation of the shape and dimensions of the total hip replacement and osseointegration implant for case 3

The implant was designed and customized by the principal investigator (MAM) based on computed tomography scans and radiographs. Each implant comprised five components (Fig. 2). The first was a hip joint prosthesis consisting of the acetabular cup (SUNFIT TH 69—dual mobility acetabular cup, Novae® range, SERF, France), which was a cementless titanium shell with an ultra-high molecular weight polyethylene liner. The second was a 28-mm ceramic head (Biolox Forte). The third was a modified 135° modular proximal body revision hip implant made of cobalt chrome with a spongiosa coating (Orthodynamics, Lubeck, Germany). The fourth was a custom-made titanium stem with sharp fins placed proximally and interspersed with plasma spray. This stem allowed connection of the proximal body to the final component that was a standard, highly polished, dual-cone adaptor coated with niobium oxide.

### Surgery stage I

The procedure was performed under a spinal anaesthetic, and 2 g of cephazolin antibiotic was administered intravenously prophylactically. The hip was exposed using the direct anterior approach [26]. An anterior capsulotomy was performed followed by dislocation of the hip joint to preserve the femoral head. Neck osteotomy was performed using a reciprocating saw, and the femoral head/neck was used as a structural autograft, adding length to the femoral residuum distally. The acetabulum was prepared by sequential reaming up to bleeding cancellous bone, which was followed by press-fit implantation of an appropriately sized acetabular shell. The sciatic nerve was identified, and any associated neuroma was excised.

**Fig. 3:**
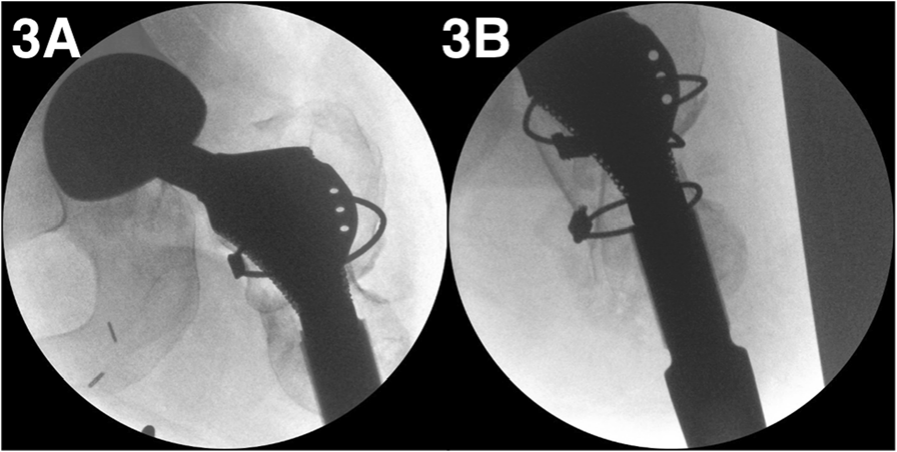
Examples of intraoperative radiographs for case 3, 3A antero-posterior view, 3B lateral view

Subsequently, the femoral canal was prepared by gentle broaching. The proximal body revision stem was then press-fitted in an antegrade fashion. The modular stem was passed through the excised femoral head to add length to the residual femur. The modular stem was then mechanically connected to the proximal body in a distal position via an opening in the distal femur, and a trial femoral head prosthesis was inserted. The hip joint was subsequently reduced and taken through a range of motion to assess stability, and the positions were confirmed using an image intensifier (Fig. 3). This was followed by implantation of the definitive femoral head implant. The soft tissue was refashioned followed by reorganization of the muscles around the proximal femur that were attached to the femoral head autograft using intraosseous non-absorbable fibre-wire sutures. The femoral construct was reinforced by Dall-Miles cable placement around the graft. The subcutaneous tissue was reduced, closing the skin nearest the distal end of the implant, and two drains were inserted.

### Surgery stage II

A guidewire was inserted distally to identify the centre of the implant under image intensifier guidance. A stoma was created using a cannulated coring device passed over the guidewire through the skin. Haemostasis was achieved at the stoma site. A dual-cone adapter was inserted in the distal end of the femoral component and locked in place using a screw.

### Postoperative care

Wound care involved twice-daily dressing changes using dry ribbon gauze. Sutures were removed 18–21 days after surgery. Once the wound had granulated, patients were instructed to wash their stoma with soap and warm water and pat dry [25].

### Rehabilitation

The rehabilitation process commenced on the day after surgery. The Osseointegration Group of Australia Accelerated Protocol was implemented, which was developed using both the THR and osseointegration fixation guidelines [27]. Non-weight-bearing mobilization, using a forearm support to gradually increasing hip motion range, and core strengthening exercises were initiated on postoperative day 3. Following the second stage of surgery, a progressive loading regime was commenced as described previously [25].

### Outcomes

Clinical and functional outcomes were measured at baseline and a minimum of 1.5-year follow-up. Plain radiographs were conducted at baseline, 3 months, 6 months, 12 months, and each year thereafter. At follow-up, the stoma was monitored for discharge and granulation, and any adverse events were recorded.

Functional outcomes were assessed using the physical and mental components of Short Form 36 (SF-36) health survey and the Questionnaire for Transfemoral Amputees (Q-TFA) [28, 29]. Ambulation ability was assessed at baseline and at 6 and 12 months using the standard Timed Up and Go (TUG) test and 6-min walk test (6MWT). The Medicare Functional Classification Level was used to classify mobility, measured as ‘K-levels’, which were graded from K-0 (no ambulatory ability) to K-4 (active adult) [30].

### Data analyses

The differences between follow-up and baseline values were calculated in measurement units and as a percentage of the baseline value. A Wilcoxon test was used to test for statistically significant differences. A *p* value of <0.05 was considered significant. Analyses were conducted using the SPSS statistical software package (Version 22).

## Results

### Patient characteristics

Patient characteristics are shown in Table 1. Patients included two females and one male, aged between 35 and 65 years. All patients had a short residuum; case 1 was 7 cm, case 2 was 9 cm, and case 3 was 3.5 cm. All cases were diagnosed with severe osteoporosis, and cases 2 and 3 had severe hip arthritis. Two patients were wheelchair-bound, and the other, who had a customized socket, presented with walking difficulties even for short distances due to a host of socket-interface problems. At baseline, patients presented with significant flexion contractures of the affected hip joint varying from 15° for case 2, who used a socket prosthesis, to 45° for case 3. All patients complained of phantom limb pain and sensation. For all cases, stage 1 of the procedure occurred between July 2013 and July 2014. Patient follow-up ranged from 18 to 30 months.

**Table 1:** Patient baseline characteristics, amputation information, and rehabilitation timeline

Case	Demographics					Amputation				Rehabilitation timeline	
	Sex	Age (years)	Height (m)	Mass (kg)	BMI	Cause	Years since amputation	Length (cm) of residuum		Days between S1 and S2	Months S1 and follow-up
									(% SND)		
1	F	46	1.55	47	20	Trauma	12	7.1	18	–	30
2	M	65	1.75	86	28	Tumour	19	8.7	17	58	25
3	F	35	1.45	75	36	Trauma	1	3.5	7	35	18

*BMI* body mass index, *m* metres, *kg* kilograms, *SND* sound limb

### Clinical outcomes

**Fig. 4:**
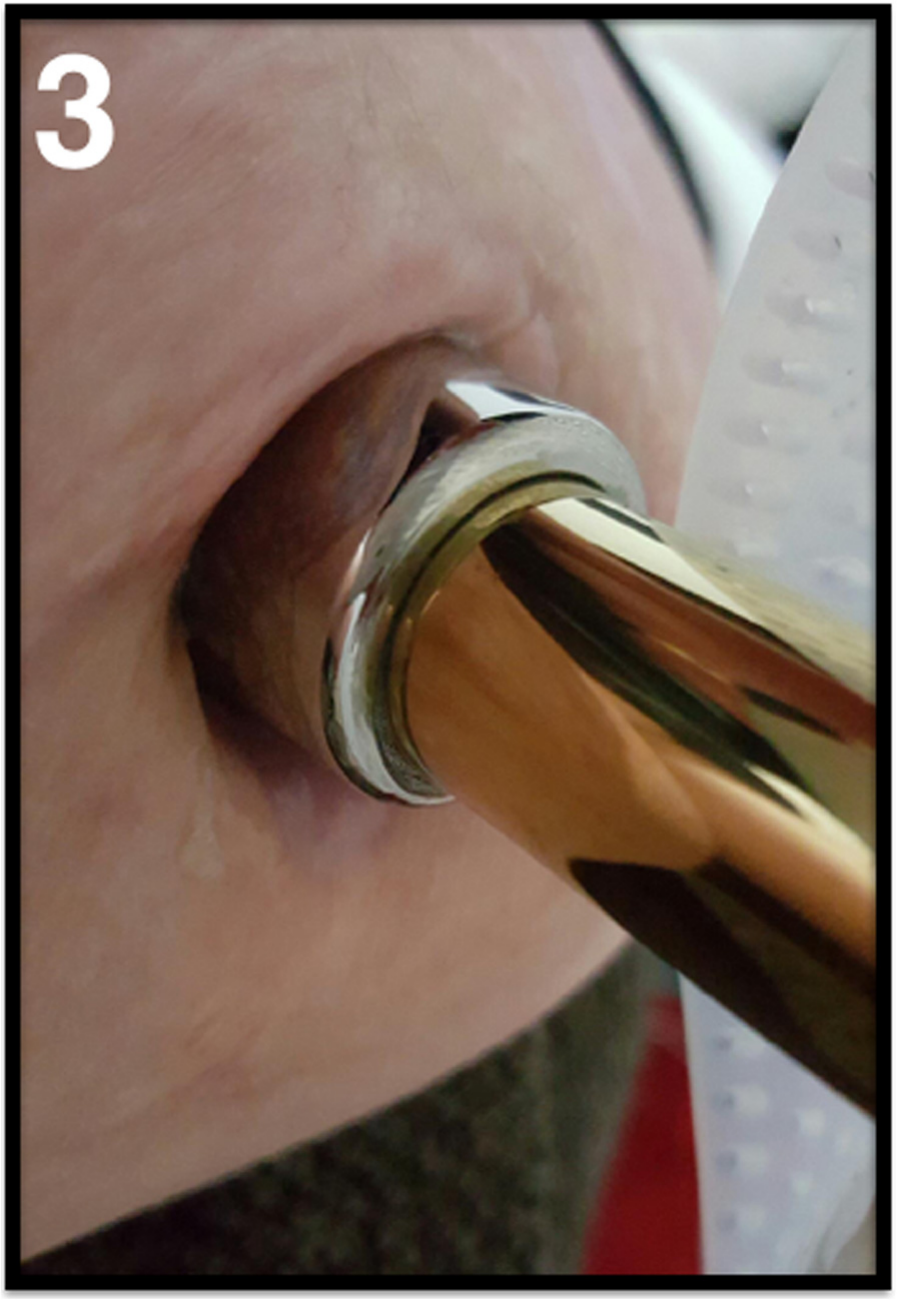
Stoma for case 3

**Fig. 5:**
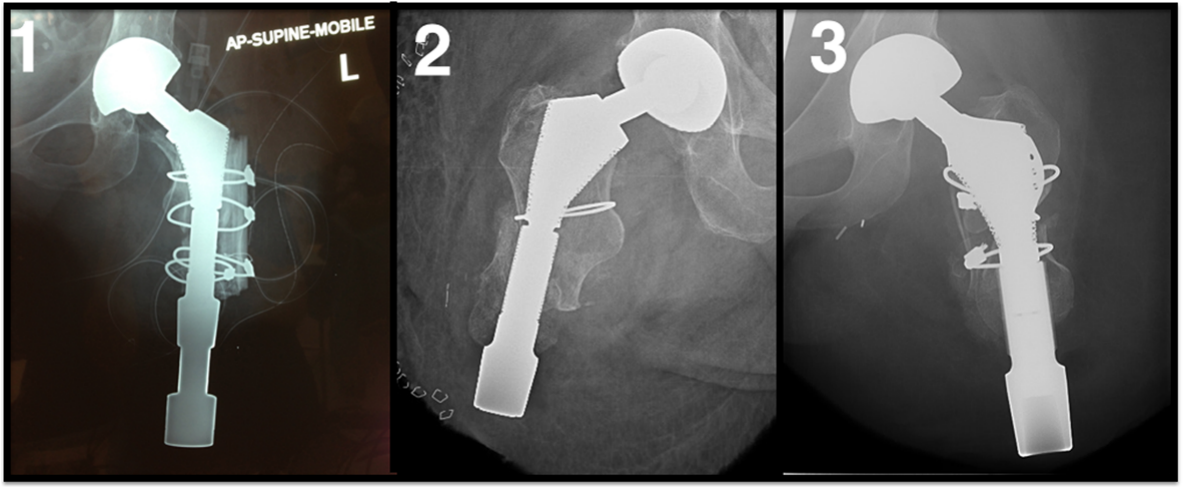
Radiographs at follow-up for case 1, 2 and 3

All patients had a pain-free hip and a normal hip motion range at follow-up. The phantom limb sensation was reduced in all three cases. All patients showed complete healing at the 3-month follow-up (Fig. 4). None of the cases presented with tissue granulation. One case (case 2) had a single episode of superficial infection that was treated successfully with a week-long course of oral antibiotics (cephalexin 500 mg). All implants were stable and well-aligned (Fig. 5).

### Functional outcomes

All patients demonstrated improvement at follow-up for both the physical (score improvement range, 2–16 points) and mental (score improvement range, 2–40 points) components of the SF-36. All patients showed improvement in the Q-TFA (score improvement range, 26–42 points), although the difference was not statistically significant (*p* = 0.11). All cases had improved mobility. On functional testing, they could cover >200 m in the 6MWT and performed TUG in <15 s (Table 2).

**Table 2:** Health-related quality of life and functional outcomes at baseline and 18–30 months follow-up

Case	Health-related quality of life									Mobility predictor			Ambulation tests					
	SF-36						Q-TFA (points)			K-levels (K0–K4)			TUG (s)			6MWT (min)		
	PCS (points)			MCS (points)														
	pre	post	diff	pre	post	diff	pre	post	diff	pre	post	diff	pre	post	diff	pre	post	diff
1	26	42	16	29	69	40	40	67	27	0	3	3	WB	12.7	–	WB	206	–
2	43	50	7	51	62	11	42	75	33	2	3	1	14	10.5	−3.5	210	540	330
3	45	47	2	63	65	2	33	75	42	0	3	3	WB	13	–	WB	200	–

*Diff* difference between baseline (preoperative) and follow-up (postoperative), *SF*-*36* Short Form 36 health survey, *PCS* physical condition summary, *MCS* mental condition summary, *Q*-*TFA* Questionnaire for Transfemoral Amputees, *TUG* Timed Up and Go, *6MWT* 6-min walk test, *WC* wheelchair-bound, *SD* standard deviation

## Discussion

We reported the feasibility of the first three attempts to combine an osseointegrated femoral implant with THR for prosthetic attachment in transfemoral amputees with a short residuum and concomitant osteoporosis.

We are not aware of any alternative techniques for rehabilitation for this group of patients. In these patients, the prosthetic function is usually poor because of reduced muscular control, improper alignment of sockets resulting from the small surface area, and compounded socket-skin interface problems [3, 18]. Consequently, the majority of these patients are confined to a wheelchair because of significantly disabling socket-interface problems when using a standard prosthesis. As is the case for transtibial amputees, a more proximal amputation is not an alternative option because hindquarter amputation would have a substantially negative effect on patient mobility and quality of life [18].

Existing reports suggest the efficacy of osseointegrated implants for transfemoral amputees [14, 25, 31]. However, the patient group described here was not considered candidates for osseointegrated implants because observational data have indicated that the length of the residuum has a significant influence on the outcome; short residuums were associated with a high risk of failure [32]. The presence of osteoporosis is also a contraindication, and data on the use of standard techniques in these patients are lacking.

This novel concept of combining joint replacement with osseointegrated implants was based on involving the proximal joint in implant fixation to provide the necessary surface area for bone-implant contact and subsequent osseointegration. Such an approach allows direct transmission of weight across the joint to the proximal bone, as we demonstrated recently in transtibial amputees [25]. Therefore, this technique not only overcomes the biomechanical challenges in these patients but also restores proximal joint motion in those with arthritis. In the present study, all three cases demonstrated significantly improved functional outcome and mobility. Moreover, the two patients who were confined to a wheelchair before the surgery regained the ability to walk.

To warrant ongoing study of this technique, the clinical benefits observed must outweigh the potential harms. The concept of a bone-anchored metal implant, which may protrude through the skin, raises serious concerns about the risk of ascending infection and its related local and systemic implications, more so than that associated with joint replacement. Therefore, careful soft-tissue management techniques and initial press-fit implantation of the osseointegrated implant are essential to provide a substantial seal to prevent the ascent of infection. Additionally, in our experience, when compared to the short tibial residuum, THR with osseointegration in the short proximal femur presents further surgical challenges related to a higher soft-tissue volume, lack of muscle mass, and low BMD. Therefore, there might be an increased potential for deep infection and subsequent joint infection in this procedure, which remains our foremost concern.

There were no serious adverse events in the present study, although there was one case of superficial infection that was treated using antibiotics. We have recently completed a multicentre study of 86 patients with transfemoral amputations who received osseointegrated implants [16]. A minimum 2-year follow-up revealed that 24 patients experienced a superficial infection that did not require surgery, whereas 5 patients had a deep infection that required soft-tissue debridement. A recent 5-year prospective study (*n* = 39) reported a <2 % incidence of deep infection leading to implant removal [15], whereas another study (*n* = 51) showed a 50 % cumulative incidence of superficial infections at a 24-month follow-up [5]. However, surgical closing techniques have been developed by us to minimize the risk of deep implant-related infection.

The present study has limitations such as its small sample size and short follow-up duration. However, we have developed a database for comprehensive data collection to enable an assessment of procedural risks and benefits and to identify potential predictors of treatment failure. We envisage that these data might facilitate prospective studies to establish further evidence-based treatment protocols. Longitudinal studies involving a larger cohort over an extended follow-up period are needed to evaluate the long-term outcomes of this novel technique.

## Conclusions

This proof-of-concept study demonstrated the feasibility of this novel surgical technique as a potential management option for transfemoral amputees with a short residuum and osteoporosis, who are unable to tolerate a conventional socket prosthesis or a standard osseointegrated implant. It also provides preliminary evidence concerning the safety and functional benefits of the procedure, but larger prospective studies are essential to establish the safety and effectiveness of the technique.

### Ethics approval and consent to participate

The present study was conducted at the clinics of A/Prof Munjed Al Muderis at Norwest Private Hospital. The Human Research Ethics Committee at the University of Notre Dame, Australia, approved this study (Reference number: 014153S). Patient data, including all clinical data and images, were used after obtaining the patients’ permission.

## Abbreviations

6MWT: 6-min walk test
BMD: bone mineral density
Q-TFA: Questionnaire for Transfemoral Amputees
SF-36: Short Form-36
THR: total hip replacement
TUG: Timed Up and Go
